# Remote Ischaemic Conditioning in STEMI Patients in Sub-Saharan AFRICA: Rationale and Study Design for the RIC-AFRICA Trial

**DOI:** 10.1007/s10557-021-07283-y

**Published:** 2021-11-05

**Authors:** Kishal Lukhna, Derek J. Hausenloy, Abdelbagi Sidahmed Ali, Abdullah Bajaber, Alistair Calver, Arthur Mutyaba, Awad Abdalla Mohamed, Brian Kiggundu, Chishala Chishala, Ebrahim Variava, Ehab Ali Elmakki, Elijah Ogola, Eltayeb Hamid, Emmy Okello, Isam Gaafar, Keiran Mwazo, Makoali Makotoko, Mergan Naidoo, Mohamed Elhadi Abdelhameed, Motasim Badri, Nasief van der Schyff, Omaima Abozaid, Paul Xafis, Sara Giesz, Trevor Gould, Waldo Welgemoed, Malcolm Walker, Mpiko Ntsekhe, Derek M Yellon

**Affiliations:** 1 Division of Cardiology, Groote Schuur Hospital and Faculty of Health Sciences, University of Cape Town, Cape Town, South Africa; 2 The Hatter Cardiovascular Institute, University College London, London, UK; 3 Cardiovascular & Metabolic Disorders Program, Duke-National University of Singapore Medical School, Singapore, Singapore; 4 National Heart Research Institute Singapore, National Heart Centre, Singapore, Singapore; 5 Yong Loo Lin School of Medicine, National University Singapore, Singapore, Singapore; 6 Cardiovascular Research Center, College of Medical and Health Sciences, Asia University, Taichung, Taiwan; 7 Sudan Heart Centre, Khartoum, Sudan; 8 Mombasa hospital, Mombasa, Kenya; 9 Tshepong Hospital, Klerksdorp, South Africa; 10 Division of Cardiology, Charlotte Maxeke Johannesburg Academic Hospital and University of Witwatersrand, Johannesburg, Gauteng South Africa; 11 Al Shaab Teaching Hospital, Khartoum, Sudan; 12 Royal Care International Hospital, Khartoum, Sudan; 13 Uganda Heart Institute, Kampala, Uganda; 14 Division of Cardiology, Greys Hospital and University of KwaZulu Natal, Pietermaritzburg, South Africa; 15 Aliaa Specialist Hospital, Omdurman, Sudan; 16 Kenyatta National Hospital, Nairobi, Kenya; 17 Omdurman Accident and Emergency Hospital, Khartoum, Sudan; 18 Coast General Teaching Hospital, Mombasa, Kenya; 19 Division of Cardiology, Universitas Academic Hospital, Bloemfontein, South Africa; 20 Division of Family Medicine, Wentworth Hospital, University of KwaZulu Natal, Durban, South Africa; 21 Al Saha Specialised Hospital, Khartoum, Sudan; 22 Department of Epidemiology and Biostatistics, King Saud Bin Abdulaziz University for Health Sciences, University of Riyadh, Riyadh, Saudi Arabia; 23 Victoria Hospital, University of Cape Town, Cape Town, South Africa; 24 Medani Heart Centre, Wad Medani, Sudan; 25 Department of Medicine, George Hospital, George, South Africa

**Keywords:** ST elevation myocardial infarction, Cardioprotection, Remote ischaemic conditioning, Ischaemia/reperfusion injury, Hospitalization for heart failure

## Abstract

**Purpose:**

Despite evidence of myocardial infarct size reduction in animal studies, remote ischaemic conditioning (RIC) failed to improve clinical outcomes in the large CONDI-2/ERIC-PPCI trial. Potential reasons include that the predominantly low-risk study participants all received timely optimal reperfusion therapy by primary percutaneous coronary intervention (PPCI). Whether RIC can improve clinical outcomes in higher-risk STEMI patients in environments with poor access to early reperfusion or PPCI will be investigated in the RIC-AFRICA trial.

**Methods:**

The RIC-AFRICA study is a sub-Saharan African multi-centre, randomized, double-blind, sham-controlled clinical trial designed to test the impact of RIC on the composite endpoint of 30-day mortality and heart failure in 1200 adult STEMI patients without access to PPCI. Randomized participants will be stratified by whether or not they receive thrombolytic therapy within 12 h or arrive outside the thrombolytic window (12–24 h). Participants will receive either RIC (four 5-min cycles of inflation [20 mmHg above systolic blood pressure] and deflation of an automated blood pressure cuff placed on the upper arm) or sham control (similar protocol but with low-pressure inflation of 20 mmHg and deflation) within 1 h of thrombolysis and applied daily for the next 2 days. STEMI patients arriving greater than 24 h after chest pain but within 72 h will be recruited to participate in a concurrently running independent observational arm.

**Conclusion:**

The RIC-AFRICA trial will determine whether RIC can reduce rates of death and heart failure in higher-risk sub-optimally reperfused STEMI patients, thereby providing a low-cost, non-invasive therapy for improving health outcomes.

## Introduction

Acute myocardial infarction (AMI) and the heart failure (HF) that often follows are among the leading causes of death and disability in sub-Saharan Africa (SSA) and worldwide [1]. The prevalence of ischaemic heart disease and its related mortality rate is predicted to rise by 70% in African men and 74% in women by 2030, and the burden of acute coronary syndromes in sub-Saharan Africa are steadily rising [2, 3]. Observational studies in sub-Saharan Africa have reported high in-patient mortality rates in STEMI patients ranging from 15 to 21%, with unacceptably high post-myocardial infarction (MI) heart failure rates [1, 2].

In most high-income countries, patients presenting with an acute ST-segment elevation myocardial infarction (STEMI) undergo immediate revascularization by primary percutaneous coronary intervention (PPCI) as the treatment of choice. However, in many low-and middle-income countries in sub-Saharan Africa, the availability and access to modern, expensive infrastructure and interventions such as PPCI are limited, and this is unlikely to change in the near future. As a result, of the STEMI patients who receive reperfusion, the majority are treated by thrombolysis and are more likely to develop heart failure and death post-STEMI, highlighting a higher-risk patient population. As such, there remains an urgent unmet need to discover novel therapeutic interventions that will improve clinical outcomes and prevent the onset of heart failure following STEMI.

In this regard, remote ischaemic conditioning (RIC), an endogenous cardioprotective phenomenon in which brief cycles of sublethal ischaemia and reperfusion applied to the limb, has been shown to reduce MI size in small and large animal models of acute myocardial ischaemia/reperfusion injury [4]. Crucially, the cardioprotective stimulus of RIC can be induced by simply inflating and deflating a pneumatic cuff placed on the upper arm or thigh for four 5-min cycles as adjunctive therapy in STEMI patients, making RIC an attractive low-cost and non-invasive treatment strategy for improving clinical outcomes in STEMI patients [5–10]. Although several clinical studies of STEMI patients reported reduced MI size with RIC, the large European CONDI-2/ERIC-PPCI multi-centre, randomized, clinical trial did not find any improvement in clinical outcomes [11]. Potential reasons for this failure to translate RIC into patient benefit included the low-risk population recruited into that study and the fact that patients who had received optimal reperfusion therapy by PPCI contributed to an unexpectedly low event rate in the sham treatment group, resulting in an unpowered trial [12–14]. In the RIC-AFRICA trial, we investigate whether RIC can improve clinical outcomes in higher-risk STEMI patients in sub-Saharan Africa treated by thrombolysis.

## Methods

### Study Design

The RIC-AFRICA study is a sub-Saharan African prospective, multi-centre, randomized, phase 3 double-blinded, sham-controlled clinical trial of 1200 STEMI patients across 20 sites in South Africa, Kenya, Sudan, and Uganda. Patients who are ineligible because they present after 24 h will be concurrently recruited into an independent observational arm of the study if they are within 72 h of MI onset. The study will be conducted in accordance with the Declaration of Helsinki and has received approval from the ethics committee of the University of Cape Town (September 10, 2021/ HREC107.2021). Trial enrolment will begin from December 01, 2021, and extend over a 24-month period. The RIC-AFRICA study protocol has been registered at www.clinicaltrials.gov (identifier: NCT04813159), and the study will be conducted and reported according to the CONSORT statement [15]. An independent data and safety monitoring board has been selected to oversee the trial. All participants will provide informed written consent.

### Study Population

We will be recruiting 3 different strata of STEMI patients.

1. Adult patients (≥ 18 years old) presenting with STEMI receiving thrombolytic therapy within guideline-recommended time (i.e., within ±12 hours of most severe chest pain)
2. Adult patients (≥ 18 years old) presenting with STEMI who are ineligible for thrombolysis because they present outside of guideline-recommended time (± 12 h) but within 24 h of most severe chest pain
3. Adult patients (≥ 18 years old) presenting with evidence of STEMI who do not receive thrombolysis and who present ≥ 24 h and within 72 h of most severe chest pain

### Interventional Arm: Randomized Control Trial

Patients who are deemed eligible for randomization into the trial on account of presentation with STEMI within 24 h will be eligible for the interventional arm of the study if the following inclusion/exclusion criteria are met.

#### Inclusion Criteria

I. Adult patients (≥ 18 years old) presenting with suspected STEMI (new ST-elevation at the J-point in two contiguous leads with the cut-points: ≥ 0.1mV in all leads other than V2-3 where the following cut-points apply: ≥0.2mV in men ≥40 years (≥0.25 mV in men <40 years) or ≥ 0.15mV in women)
II. Within 24 h of onset of myocardial infarction as deemed by the attending clinician
III. Signed informed consent

#### Exclusion Criteria

I. STEMI patients undergoing primary percutaneous coronary intervention
II. STEMI patients presenting with cardiogenic shock or haemodynamic instability (systolic blood pressure (SBP) < 90 mmHg for ≥ 30 min, or use of pharmacological and/or mechanical support to maintain SBP ≥ 90 mmHg, and evidence of end-organ damage such as urine output of < 30 mL/h, altered mental status, and/or serum lactate > 2.0 mmol/L) [16]
III. Contra-indication to thrombolytic therapy in patients presenting within guideline-recommended time (± 12 h)
IV. Conditions that preclude the use of RIC or sham-control on either arm such as:
  - Severe active skin disease/burns on both arms
  - Bilateral upper limb amputations
  - Evidence of acute limb ischaemia on either arm
  - Active upper limb gangrene of any digits
  - Bilateral arteriovenous fistulae needed for haemodialysis
  - Known breast cancer with ipsilateral lymph node involvement on the side of RIC
V. Inter-current disease with an expected life expectancy of less than 24 h

### Observational Arm

There is currently very limited data surrounding late STEMI presentation, management, and outcomes across Africa where the condition is thought to be common. As this will be one of the first large scale interventional STEMI trials conducted across Africa, we have decided to use this opportunity to gather as much information as possible about patients who present with late STEMI to help narrow the gap on this subject in the medical literature, while concurrently generating future research opportunities in Africa. The participants in the observational arm will not be compared to the participants randomized to the trial intervention.

Patients who are deemed ineligible for randomization into the clinical trial on account of presentation beyond 24 h will be eligible for the observational arm of the study if the following inclusion/exclusion criteria are met.

#### Inclusion Criteria

I. Signed informed consent
II. Adult patients (≥ 18 years old) with clinical evidence of STEMI older than 24 h and less than 72 h as defined by the following:
  - Compatible history with maximal chest pain between 24 and 72 h prior to presentation
  - Compatible biomarkers (elevated cardiac troponin)
  - ECG compatible with recent STEMI; and/or
  - Compatible echocardiography

#### Exclusion Criteria

I. Refusal or inability to sign informed consent

### Randomization

Consenting participants who present within 24 h of MI onset will be randomized to receive either RIC or sham control. Randomization will be conducted via an automated, secure web-based program built into the data capturing software. Randomization will be stratified according to recruiting site and time of MI-onset, using random permuted blocks. The randomization schedule will be conducted in a manner that ensures that 2 participants in stratum 1 (receives thrombolysis within ± 12 h of MI onset) are recruited for every participant in stratum 2 (outside the thrombolytic window but within 24 h of MI onset) and will ensure equal allocation to both the active and sham control arms. The patient, treating clinician, and study investigators will be blinded to the treatment allocation. Willing participants who present between 24 and 72 h of MI onset will be recruited into the concurrently running independent observational arm of the RIC-AFRICA study.

### Trial Intervention

An automated pneumatic blood pressure device (manufactured by Seagull Aps in Denmark) will be used to deliver either the RIC or sham protocol. The RIC protocol will comprise of applying the blood pressure cuff at recruitment on the upper arm to automatically deliver four 5-min alternating high-pressure cuff inflations (20 mmHg above each participant’s systolic blood pressure) and cuff deflations, such that the total RIC protocol takes 40 min. The sham control protocol will comprise the application of a visually identical pneumatic cuff on the upper arm which will automatically deliver four 5-min alternating low-pressure cuff inflations (20 mmHg) and cuff deflations, for a total of 40 min. The RIC and sham protocol will be repeated daily over the next two days. Enrolled participants in stratum 1 will receive RIC or sham control within 1 h of thrombolysis. Participants in stratum 2 will receive RIC or sham control within 1 h of randomization. The RIC and sham devices will be applied by the unblinded research nurse. None of the study interventions or procedures will interfere with standard STEMI management at each site. Participants enrolled in the observational arm will not receive any trial intervention.

### Study Endpoints

#### Primary Endpoint

For the purpose of this trial, all endpoints were consistent with the 2017 cardiovascular and stroke endpoint definitions for clinical trials consensus document [17].

The primary composite endpoints of the study will be all-cause death and early post-MI heart failure. The latter is defined as (a) pre-discharge (in-hospital) heart failure or (b) heart failure hospitalization within 30 days of discharge from index myocardial infarction (see Table 1 of Appendix for more details).

#### Secondary Endpoint

Secondary outcome measures will include a composite clinical endpoint of MACCE at 30 days follow-up, defined as (i) all-cause mortality; (ii) non-fatal myocardial infarction; (iii) transient ischaemic attack or stroke; and (iv) heart failure with or without hospitalization (see Table 1 of Appendix for more details).

### Sample Size Determination

In a recent audit of STEMI patients across several participating sub-Saharan African hospitals, we found an all-cause mortality rate of 14% and an 18% cumulative risk of readmission with post-MI heart failure requiring in-hospital treatment at 30 days. We have based our power calculations on these recent audit data, using a primary combined event rate of 30%. We have estimated the effect size to be a 25% relative risk reduction in the 30-day event rate of all-cause death and heart failure hospitalization. We selected a 25% reduction as this effect size would be clinically meaningful and achievable for such a high-risk population. To demonstrate a 25% reduction in the primary composite endpoint, we will need to recruit 1078 STEMI patients in total to achieve 80% power, at the 5% significance level. Therefore, allowing for a conservative 10% drop-out rate at 30 days, we intend to recruit 1200 STEMI patients across the 20 recruiting centres over the 24-month recruitment period. The sample size does not include participants recruited into the observational arm of the study.

### Statistical Analysis

All efficacy endpoints will be analysed according to the intention-to-treat principle. The primary analysis will be a comparison of all-cause death and post-MI heart failure hospitalization event rates at 30 days after randomization between the RIC and sham control arms in all randomized STEMI participants. Differences in means among the randomized groups will be analysed using the analysis of variance (ANOVA) test and, if required, the Turkey post hoc test, and differences in proportions using the χtest, or Fisher exact test, as appropriate. Follow-up will be measured from date of randomization until date of occurrence of the composite event (death or heart failure for primary analysis, and death, non-fatal MI, transient ischaemic attack or stroke, or HF with or without hospitalization for the secondary analysis) or end of the study. The primary outcomes will also be analysed in prespecified subgroups defined in our statistical analysis plan. Survival curves of study groups will be compared using the Kaplan-Meier method, and significance will be assessed using the Log-rank test. Cox proportional hazards regression models will be fitted to identify factors associated with the hazard of occurrence of the composite event. Strength of association will be expressed as a hazard ratio, with 95% confidence intervals and Wald test *p* value. All tests will be two-sided, and a *p* value < 0.05 will be considered significant. The Stata (TX, USA) statistical software will be used for data analysis.

### Clinical Study Monitoring and Data Management

The Mayosi Collaborative Clinical Trials Unit at the University of Cape Town, South Africa, and the Hatter Cardiovascular Institute at the University College London, UK, will oversee the trial.

## Discussion

In the RIC-AFRICA study, we intend to investigate the role of RIC as a cardioprotective strategy in patients presenting with STEMI in sub-Saharan Africa. RIC, an endogenous phenomenon achieved by using a blood pressure cuff that is simply inflated and deflated for four 5-min cycles, activates many neuro-hormonal prosurvival signalling pathways that mediate cardioprotection and preservation of left ventricular function in many small and large animal studies [19–22]. Although the actual mechanisms through which RIC exerts myocardial protection from a remotely conditioned arm remains unclear, there are a number of small proof-of-concept clinical studies that have reported benefits of RIC improving myocardial salvage after acute myocardial infarction [8, 23]. Unfortunately, the translation of RIC into clinical practice has been faced with many challenges and obstacles with negative benefits in myocardial ischaemia/refusion injury post-cardiac surgery [24, 25]. In the recent large CONDI-2/ERIC-PPCI trial, RIC was unable to improve clinical outcomes in low-risk, uncomplicated STEMI patients recruited in high-income countries that were optimally treated by PPCI [11]. The CONDI-2/ERIC-PPCI trial recruited mainly a low-risk patient population as evidenced by the following: (i) the low cardiac mortality rate of 2.7% at 12 months; (ii) 96% of the cohort having presented without symptoms or signs of heart failure (Killip class I); (iii) the median acute MI size assessed by cardiac MRI in the first week following PPCI was relatively small, with a median MI size of 17% of left ventricular mass; (iv) the total acute myocardial ischaemia time was short with a median symptom onset to PPCI time of only 3 h; and finally, (v) the prevalence of cardiovascular risk factors were relatively low with 40% of patients having a history of hypertension, and only 10% having medically treated diabetes [13]. The benefit of RIC in higher-risk MI patients with greater comorbidities, larger infarct sizes, and longer total ischaemic times remains unknown.

The RIC-AFRICA study is particularly timely as the burden and mortality rates following STEMI experienced in low-and middle-income countries in sub-Saharan Africa are on the rise despite a decline noted globally [2, 3]. Other factors contributing to the observed worse outcomes in STEMI patients in sub-Saharan Africa include the following: (i) inadequate access to hospital facilities, especially in rural areas, resulting in prolonged transfer times to facilities where thrombolytic treatment is available [26]; (ii) the increased prevalence of cardiovascular risk factors such as hypertension (in up to 60% of patients) and diabetes (in up to 40% of patients), which remains undiagnosed and untreated in many people [26]; (iii) sub-optimal use and compliance of secondary preventative guideline-directed medical therapy at discharge post-STEMI; and (iv) high total acute myocardial ischaemia times with delayed presentations (> 6 h of chest pain onset) in nearly 70% of patients, confirming a higher-risk population [1].

Preliminary evidence on the safety, feasibility, and potential cardioprotective efficacy of RIC in STEMI patients treated by thrombolysis have already been demonstrated in the previously published phase 2, multi-centre, randomized controlled ERIC-LYSIS trial in the Island of Mauritius in which RIC initiated prior to thrombolysis reduced MI size as measured by a 32% reduction in 24-h area-under-the-curve serum cardiac biomarkers [23]. However, as recently discussed [12], there remains a need for a prospectively designed, randomized, sham-controlled clinical trial to determine whether RIC can improve clinical outcomes in high-risk patient groups.

Despite RIC failing to improve clinical outcomes in low-risk STEMI patients undergoing modern reperfusion therapy with primary PCI [11], the cardioprotective efficacy of RIC in higher-risk STEMI patients treated by thrombolysis in low-and middle-income countries where PPCI is not widely available remains largely unknown and potentially an attractive, more efficacious, low-cost, and non-invasive therapeutic solution.

In summary, the RIC-AFRICA trial will determine whether the simple, low-cost, non-invasive intervention of RIC can improve clinical outcomes in higher-risk STEMI patients treated by thrombolysis.

## Data Availability

As this is an announcement paper, no original data has been generated.

## Appendix

**Table 1.** Definition of study endpoints according to the 2017 cardiovascular and stroke endpoint definitions for clinical trials [17]

Pre-discharge heart failure	I. New or worsening symptoms of heart failure (dyspnoea, decreased exercise tolerance, fatigue, or symptoms of worsened end-organ perfusion or volume overload) II. Objective clinical evidence of heart failure and fluid retention (such as pulmonary crepitations/crackles, raised JVP, S3 gallop, peripheral oedema, increasing abdominal distension/ ascites, or rapid weight gain thought to be related to fluid retention) III. Radiological evidence of pulmonary congestion/ oedema IV. Increased cardiac biomarkers (such as BNP > 500 pg/mL or NT-proBNP > 2,000 pg/mL) V. The initiation of intravenous or the significant augmentation of oral loop diuretic therapy
Heart failure hospitalization at 30 days	I. An admission ≥ 24 h II. A diagnosis of heart failure made by the treating clinician III. The initiation of intravenous or the significant augmentation of oral loop diuretic therapy
Non-fatal myocardial infarction	Acute re-infarction MI that occurs in the first 28 days after the index admission for STEMI, defined by [18]: I. Recurrent ischaemic symptoms II. New ST segment deviation in at least two contiguous leads III. A ≥ 20% increase between an immediate cTn value taken at re-admission and a subsequent cTn value taken 3–6 h later, exceeding the 99th percentile upper range limit
Transient ischaemic attack	A transient episode of focal neurological dysfunction caused by brain, spinal cord or retinal ischemia, without acute infarction
Stroke	An acute episode of focal or global neurological dysfunction caused by brain, spinal cord, or retinal vascular injury as a result of haemorrhage or infarction
Heart failure without hospitalization	An event that meets the clinical criteria for heart failure but does not meet the length-of-hospital stay, i.e. an admission < 24 h

## Abbreviations

AMI: Acute myocardial infarction
HF: Heart failure
MACCE: Major adverse cardiac and cerebrovascular events
MI: Myocardial infarction
PCI: Percutaneous coronary intervention
PPCI: Primary percutaneous coronary intervention
RCT: Randomized control trial
RIC: Remote ischaemic conditioning
SSA: Sub-Saharan Africa
STEMI: ST elevation myocardial infarction
cTn: Troponin
